# Opportunities to Improve Nutrition for Patients in Hospital After Discharge From an Intensive Care Unit: A Human Factors Analysis

**DOI:** 10.1111/nicc.70374

**Published:** 2026-02-01

**Authors:** Sarah Vollam, Owen Gustafson, Lauren Morgan, Natalie Pattison, Hilary Thomas, Peter Watkinson

**Affiliations:** ^1^ Nuffield Department of Clinical Neurosciences University of Oxford Oxford UK; ^2^ NIHR Oxford Biomedical Research Centre Oxford UK; ^3^ Oxford Allied Health Professions Research & Innovation Unit, Oxford University Hospitals NHS Foundation Trust Oxford UK; ^4^ Nuffield Department of Surgical Sciences University of Oxford Oxford UK; ^5^ School of Health and, Medicine and Life Sciences University of Hertfordshire Hatfield UK; ^6^ East and North Herts NHS Trust Stevenage UK; ^7^ Centre for Research in Public Health and Community Care, School of Health, Medicine and Life Sciences University of Hertfordshire Hatfield UK; ^8^ Adult Intensive Care Unit, Oxford University Hospitals NHS Foundation Trust Oxford UK

**Keywords:** enteral feeding, human factors, ICU recovery, mixed methods, nutrition

## Abstract

**Background:**

Nutrition during hospitalisation following critical illness is fundamental to rehabilitation, but provision is often poor.

**Aim:**

To analyse the process of delivering nutrition to post‐ICU patients on the ward.

**Study Design:**

This work forms part of a mixed methods study. In three representative UK hospitals, we conducted: a structured judgement review (SJR) of 300 patients who died following discharge from ICU; in‐depth reviews of 20 survivors and 20 deaths judged to be ‘probably avoidable’ in the SJR; and interviews with 55 patients, family members and staff about their experiences of post‐ICU ward care. We extracted nutrition provision information from the primary data. Using these data and the Functional Resonance Analysis Method (FRAM), we worked with stakeholders to map the process of delivering enteral feed to patients discharged from ICU to hospital wards.

**Results:**

The stakeholder meeting included a dietitian and a medical registrar from two of the three primary data collection sites, two researchers with knowledge of the primary data (with nursing and physiotherapy backgrounds) and a human factors facilitator. The FRAM revealed that providing enteral feeding on the ward is not a linear process, with three clusters of functions delivering distinct steps within the wider process: establishing the need for nasogastric feeding, the nasogastric placement cycle and nasogastric feed delivery. There are multiple points in these processes where failures in multi‐professional teamwork result in the absence of the required steps to move through the processes in a timely manner. In particular, the process for confirming nasogastric tube placement risked system‐related delays to feed administration, significantly affecting the volume of feed delivered to patients.

**Conclusions:**

The FRAM identified multiple process problems affecting nutritional support that may have led to profound consequences for post‐ICU patients, with multi‐professional collaboration a key factor for effective delivery of timely enteral nutrition.

**Relevance to Clinical Practice:**

Improving collaborative working processes and addressing common nutritional support problems after ICU discharge could improve nutritional delivery and expedite recovery from critical illness.

**Trial Registration:**

ISRCTN14658054

## Impact Statement

- What is known about the topic
  ○ Nutrition during hospitalisation following critical illness is fundamental to rehabilitation and recovery.
  ○ Previous studies have identified that nutrition provision following critical illness is poor on hospital wards.
  ○ In our previous retrospective case record review, we identified nutrition delivery as a significant contributory factor to probably avoidable deaths, with absence of a clear nutritional plan on ICU discharge common.
- What this paper adds
  ○ Mapping of the process of delivering enteral nutrition to patients on the ward following critical illness using the Functional Resonance Analysis Method (FRAM).
  ○ The FRAM identified multiple points where delays can be incurred in the process of delivering enteral nutrition.
  ○ Addressing such problems requires collaborative multi‐professional working to ensure patients receive the nutrition required to maximise their recovery from critical illness.

## 1 Introduction

Poor physical recovery affects up to 25% of patients recovering from critical illness, especially older or frail adults [1, 2]. Poor physical function may persist for years and is associated with poor quality of life and depression [3, 4]. This poor physical function is attributed to muscle loss due to catabolism during critical illness [5]. Adequate nutrition is essential for regaining muscle mass, without which patients are unlikely to regain strength and mobility following critical illness [6]. The European Society for Clinical Nutrition and Metabolism (ESPEN) describe three metabolic phases of critical illness [7]. The first two, ‘early and late acute’ phases—together lasting up to 7 days—are characterised by a catabolic stress response. During catabolism, nutrition cannot be fully utilised and muscle loss is likely, even when receiving enteral feeding [5]. During the third ‘post‐acute’ phase, anabolism may return. It is only once anabolism is re‐established that patients can utilise nutrition to support their recovery. Under this model, the post‐acute anabolic third phase will mainly occur once patients have been discharged from ICU to the ward, making this ward‐based period crucial for optimising nutrition to support physical rehabilitation [8].

## 2 Background

Three recent reviews identified that nutritional needs are often not met in the post‐ICU in‐hospital period [8, 9, 10]. Methods for overcoming nutritional under‐delivery during the recovery period following critical illness are, therefore, urgently required.

We conducted a mixed methods study of post‐ICU ward care (REFLECT: REcovery FoLlowing intensivE Care Treatment NIHR ID:PB‐PG‐0215‐36149), aiming to identify potential improvements to ward‐based care provision for critical care survivors to improve outcomes. We used three interlinked approaches (structured judgement review, in‐depth case analysis and qualitative interviews) to examine care delivery, reflecting the complex and multifactorial nature of post‐ICU ward care. The structured judgement review identified common contributory factors to probably avoidable deaths, including out‐of‐hours discharge, poor rehabilitation provision and poor nutrition management [11]. To further understand these problems and inform changes in practice to improve the recovery of critical care survivors, we used the data from the REFLECT study to map the delivery process for each of these identified problems using the Functional Resonance Analysis Method (FRAM). The FRAM methodology is an emerging technique increasingly used to understand complex processes in healthcare and understand how care or work activities take place [12]. We have successfully used this to map other areas of post‐ICU care delivery identified as problematic: out‐of‐hours discharge from ICU [13] and mobilisation [14].

## 3 Aim

Applying this approach to the identified problem of nutrition management, we aim to analyse the process of delivering nutrition to post‐ICU patients on the ward, informed by the primary data from the REFLECT study.

## 4 Design and Methods

We report our study using a combination of the Strengthening the Reporting of Observational Studies in Epidemiology (STROBE) [15] for cohort studies and the COnsolidated criteria for REporting Qualitative research (COREQ) [16] reporting guidelines, in line with our mixed methods approach.

### 4.1 Setting and Sample

We collected data at three NHS hospitals, selected to represent ICUs of different sizes and provision of post‐ICU services.

### 4.2 Definitions

We defined nutritional support as provision of total parenteral nutrition, enteral nutrition or help with oral feeding (including practical help and nutrition supplements).

Probably avoidable deaths were defined according to the Structured Judgement Review method (SJR) as those where identified problems in care resulted in harm which had a more than 50:50 chance of contributing to death [17].

### 4.3 Primary Data Collection Informing the FRAM

Under a mixed methods framework, we used three interlinked approaches to examine care delivery, reflecting the complex and multifactorial nature of post‐ICU ward care. Each approach offered complementary contextual insight into care delivery problems. To provide an overview of post‐ICU care, we reviewed the medical records of 300 patients who were discharged from ICU and subsequently died in hospital, using an established mortality review method [17]. This approach identified 20 probably avoidable deaths, and a further 65 deaths with some degree of avoidability. Common contributory factors to these probably avoidable deaths included out‐of‐hours discharge, poor rehabilitation provision and poor nutrition management [11].

To maximise the learning from these probably avoidable deaths, each common contributory factor was analysed in detail using a mixed methods approach. We reviewed each of the deaths judged probably avoidable in the SJR, alongside an equal number of survivor cases using an established framework methodology to guide identification of problems in care, and their context [18]. To broaden our understanding of the context of delivering care to this patient group, we also conducted semi‐structured interviews with 55 purposively sampled patients (*n* = 18), family members (*n* = 7) and staff (*n* = 30) about their experiences of post‐ICU ward care (further details of the participants are included in Supporting Information). Interviews were conducted by two female nurses who were experienced qualitative researchers with training in qualitative research methods. Researcher 1 had no prior relationship with any participants. Researcher 2 worked clinically in one of the sites. A proportion of interviews at that site were conducted by researcher 1 to offer an ‘outsider’ perspective. Reflective notes were taken by the researcher during and after each interview, prompting reflection on any biases, assumptions or external factors which may have affected the interview. Methods for primary data collection and analysis are reported in our published protocol [19], and in the Supporting Information along with brief results (see Supporting Information: methods for primary data collection and Tables S1–S6).

### 4.4 Ethical Approval and Study Registration

We published the protocol [19] and registered the study prospectively: ISRCTN14658054. Ethical approval was granted by Wales REC 4 (reference 17/WA/0139) on 4 May 2017.

### 4.5 Analysis: Functional Resonance Analysis Method

To develop our understanding of why nutritional problems in care occurred in this population, we used the primary data from the REFLECT study to map the delivery of nutrition to post‐ICU patients on the ward, using the FRAM [20]. This Human Factors (HF)‐based approach aims to describe the real‐world ‘work as done’ process followed to deliver the desired end goal—in this case successful delivery of nutrition to post‐ICU patients on the ward—and, crucially, the circumstances which contribute to this process. The goal is an appreciation of the complexities of the care process which may not be visible using other methods [21]. Informed by primary data [21], the method guides identification of specific activities within the process, integral to the delivery of the desired end goal (termed ‘functions’) and the circumstances needed to deliver each function (termed ‘conditions’). Conditions are further split into: inputs, preconditions, resources, time constraints and controls (Table 1).

**TABLE 1:** Definitions and examples of FRAM terms.

FRAM terms	Definition	Example
Function	Activity in a process	Nurse commences NG feed
Conditions	The circumstances needed to deliver a function—listed below	n/a
Input	Starts the function	Patient identified as needing NG feeding
Precondition	Must be satisfied before the function can start	NG aspirate confirmed at gastric contents through pH check
Resource	Needed to carry out function	NG feed pump
Control	Monitors or controls the function	Local NG guidelines
Time	Any time constraint that affects the function	Nurse time to administer feed
Output	The outcome of the function	NG feed commenced

*Note:* Adapted from Clay‐Williams et al. (2015) [20, 33].

**FIGURE 1:**
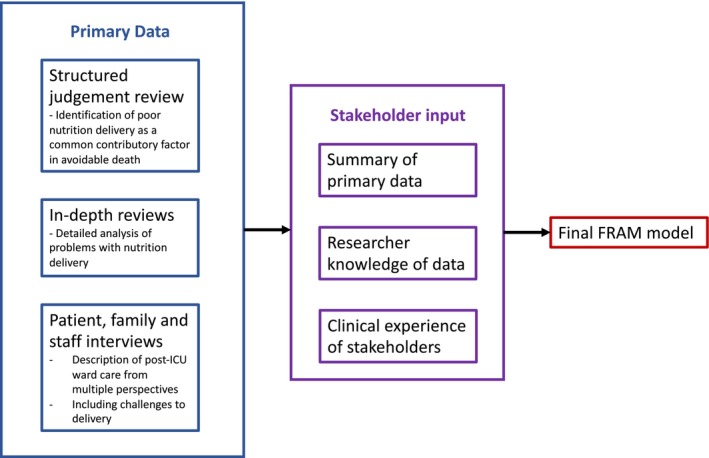
Data flow diagram.

We first presented a summary of the primary data related to the site stakeholders (see Supporting Information). We then identified the first function in the FRAM process of delivering nutrition to post‐ICU patients on the ward, written on a coloured sticky note. We discussed this function, drawing on the primary data (from the knowledge of the study team and the data summary) and the clinical experiences of the participants, prompting identification of all conditions of this function (preconditions, resources, etc.). Figure 1 describes how data informed the FRAM. We also considered whether each function and associated conditions were the same at each site, with the aim of highlighting between‐site variability. Different coloured sticky notes were used for each FRAM condition (input, precondition, etc.), and positioned around the function. This process was repeated until it was agreed that we had identified all relevant functions associated with delivering nutrition to post‐ICU patients on the ward, from both the primary data and participants' clinical experiences. We then transcribed the finalised paper‐based FRAM into the FRAM visualiser software (Version 0.4.1, May 2016: http://functionalresonance.com/FMV/index.html). The finalised FRAM is extensive, covering a wide range of functions related to nutrition delivery via various routes. For the purpose of this report, we have limited the reported activities to those related to providing enteral nutrition, as this was an area identified as particularly problematic in the in‐depth reviews and therefore having the most potential for meaningful change.

## 5 Results

Two members of the study team (a nurse and a physiotherapist, both with both critical care and ward experience) with knowledge of the primary data from the REFLECT study worked with two site expert stakeholders: a Critical Care Dietitian with ward experience at one of the sites and a Medical Specialist Registrar with ward and ICU experience at two of the sites to develop the post‐ICU nutrition FRAM. Inclusion of multiprofessional site stakeholders in development ensured face validity of the final FRAM model [21].

### 5.1 Functional Resonance Analysis—Enteral Feeding

**FIGURE 2:**
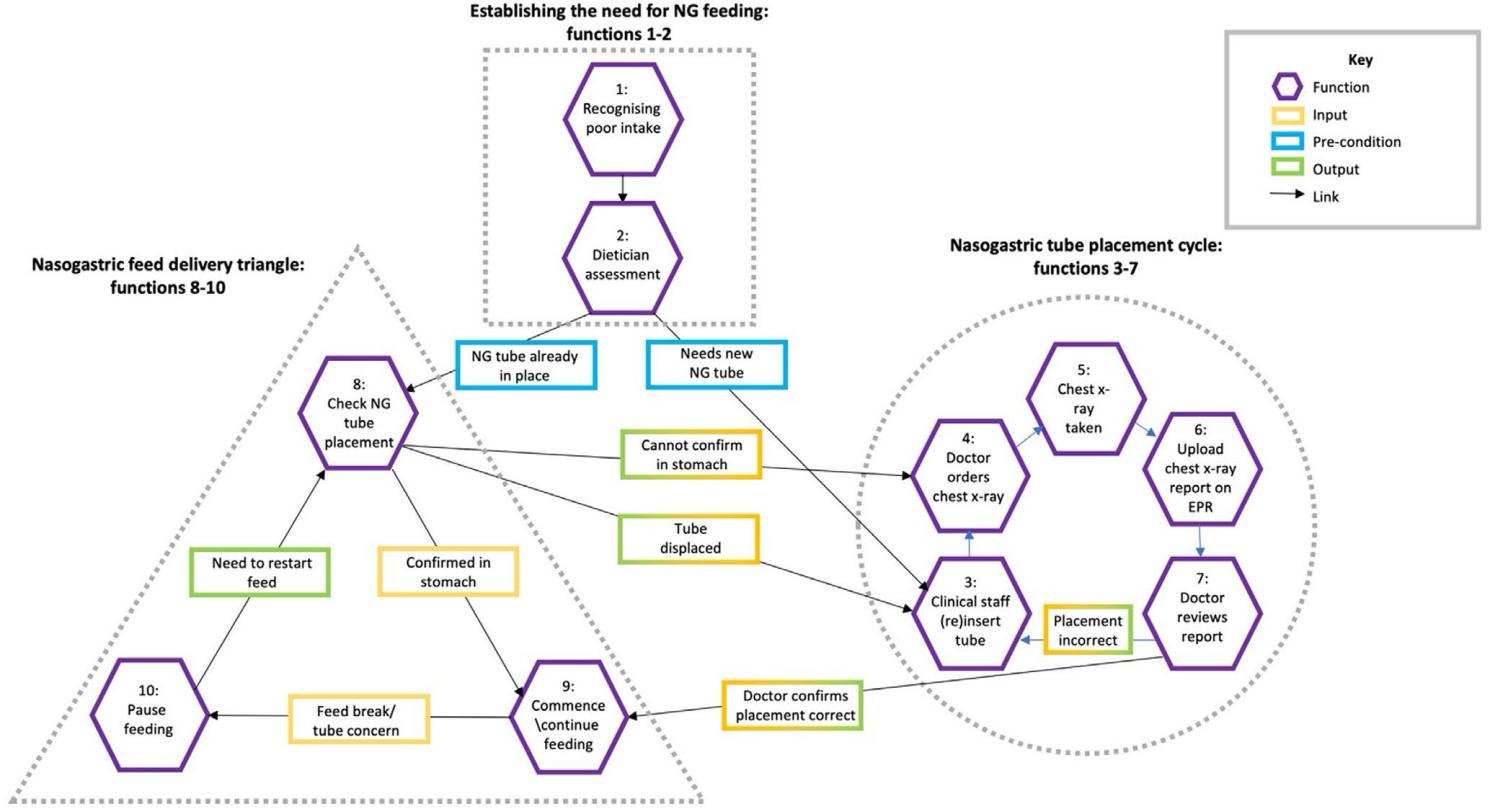
Simplified FRAM diagram of nasogastric feeding on the ward.

The process of providing enteral feeding on the ward is non‐linear. We identified 10 key functions essential to supporting adequate enteral nutrition provision on the ward, with three clusters of functions within this process: establishing the need for enteral feeding; the nasogastric (NG) tube placement cycle; and enteral feed delivery. No significant variations in functions or conditions were identified. Functions are summarised in Figure 2, with all identified conditions presented in Table 2 (functions 4–7 are summarised in Table 2 for brevity but described fully in Table S7). Outputs of one function often become inputs of the next (highlighted in Figure 2 using two‐tone boxes). We have summarised the process below.

**TABLE 2:** Output of FRAM detailing the process of providing enteral nutrition to a ward patient following discharge from ICU.

*Function 1: Recognising poor intake*	
Input:	ICU handover (NG feeding required/established) Nurse assessment Ward round assessment
Pre‐conditions	Documentation of nutritional intake Nutrition screening documented
Resources	Nurse time
Controls	Local policy on nutrition monitoring/screening
Time	Prompt assessment to prevent prolonged period without nutrition
Output	Dietitian referral
*Function 2: Dietitian assessment and feed prescription*	
Input	Dietitian referral
Pre‐conditions	Documentation of nutritional intake Blood results
Resources	Nurse time Dietitian time and skill Access to electronic notes system
Controls	Local policy NICE guidelines: CG 83, 2009 and CG 32, 2017 [22, 23]
Time	Prompt assessment to prevent prolonged period without nutrition
Output	Identified as needing support to maximise oral intake, or Identified as needing NG feeding and feed prescribed
*Function 3: Clinical staff (re)insert new nasogastric tube*	
Input	Patient requires NG tube
Pre‐conditions	Patient consent Patient condition appropriate for insertion
Resources	Clinician time and skill NG tube of correct type and size and associated equipment
Controls	Local NG tube policy
Time	n/a
Output	NG tube in place
*Functions 4–7: Radiological confirmation of tube placement*	
Input	Requirement to start NG feeding
Pre‐conditions	NG tube in place
Resources	Access to electronic patient record Team/on‐call doctor time to order chest x‐ray Radiology time to take x‐ray Porter time to take patient to department (optional) Radiologist time and expertise to review and report on x‐ray Team/on‐call doctor time to review x‐ray report and confirm placement to nurse
Controls	Local policy
Time	Timeliness of feed administration to meet nutritional needs
Output	NG tube placement in stomach confirmed Tube incorrectly placed
*Function 8: Checking placement of nasogastric tube*	
Input	Requirement to (re)start NG feeding
Pre‐conditions	NG tube in place
Resources	pH test and associated equipment Nurse knowledge and skill Nurse time
Controls	Local policy
Time	Timeliness of feed administration to meet nutritional needs
Output	NG tube placement in stomach confirmed NG tube placement not confirmed (tube dislodged/no aspirate obtained/aspirate pH test inconclusive)
*Function 9: Commence/continue feeding*	
Input	NG placement in stomach confirmed NG feed prescription
Pre‐conditions	NG tube placement confirmed
Resources	Prescribed feed Feeding pump Feeding giving set Nurse knowledge
Controls	Local policy
Time	Timeliness of feed administration to meet nutritional needs
Output	NG feed started
*Function 10: Pause feeding*	
Input	Need to pause feeding (break/NBM for procedure/placement concern)
Pre‐conditions	Communication between MDT of need to pause feeding and when NG feed prescription (if prescribed feed break) Visual check of NG tube placement (e.g., after mobilisation)
Resources	Access to electronic prescription system Nurse knowledge
Controls	Local policy
Time	Timeliness of feed pauses to reduce impact on nutritional intake
Output	NG feed paused

Abbreviations: NBM, nil by mouth; NG, Nasogastric.

### 5.2 Establishing the Need for Enteral Feeding

We identified two functions leading to prescription of enteral feeding (highlighted by the dotted square in Figure 2). Function 1—recognising a patient as having poor nutritional intake—had two possible inputs: enteral feeding may have already been established in ICU and be part of the written handover, or poor intake may be recognised on the ward by nursing or medical staff. Adequate nutritional monitoring is a pre‐condition for this function, contributing to the assessment of intake. This was absent in half of the in‐depth reviews where nutritional problems were present (see Table S3), limiting the ability of staff to recognise poor nutritional intake. Where nutritional intake was recognised as poor, the output was dietitian referral.

The output of function 1—dietitian referral—becomes the input of function 2: dietitian assessment. Resources for this function include dietitian time and access to relevant blood results. Pre‐conditions include documentation of prior nutritional intake (already identified as commonly absent). Controls include national clinical guidelines [22, 23] and local feeding guidelines. The common outputs of this function are to monitor and support oral intake or to deliver NG feeding.

### 5.3 The Nasogastric Tube Placement Cycle

We identified a cycle of functions related to placing a nasogastric tube (dotted hexagon in Figure 2). If not already in place, the clinician (usually a nurse) inserts an NG tube (function 3). Resources for this function include clinician time, skills and equipment. Pre‐conditions include appropriate patient condition and consent.

Once the tube has been sited, at all three sites the local protocol necessitates radiological confirmation of correct placement (a control). This requires several functions (4–7, dotted hexagon in Figure 2) to occur in succession—a doctor must order the chest x‐ray [4]; the chest x‐ray must be taken [5]; the report from the x‐ray must be uploaded into the electronic patient record [6]; and the doctor must review the report [7]. The main resource for each function in this cycle is staff time. Delays due to staff workload in each step of this process may cumulate into prolonged periods without nutrition. The doctor must then communicate one of two outputs to nursing staff. If the tube is incorrectly placed, this cycle returns to function 3: clinical staff (re)site tube, and the full process occurs again, incurring further delay. If the output is that the tube is correctly placed, this cycle ends and moves on to the next group of functions.

### 5.4 Nasogastric Feed Delivery

We identified three key functions in this process (8–10, dotted triangle in Figure 2). If the input is NG tube placement radiologically confirmed, the process can start at function 9 (commence feeding). If the input is NG tube already in place (and placement has not been radiologically confirmed), before feeding can be commenced/recommenced following a break, local policy at all three sites requires NG tube placement to be re‐confirmed (function 8). Placement is confirmed by visual inspection of the tube markings at entry to the nostril against documentation, and pH testing of tube aspirate (confirming the aspirate as gastric contents). Resources for this function include staff time and expertise, and equipment to conduct the test. The control for this function is local policy. If placement is confirmed, the process can move on to function 9. Alternative outputs include NG tube dislodged or visibly moved; no aspirate obtained; and pH not within range. These outputs require a return to the placement confirmation cycle, either at function 3 (clinical staff (re)insert tube) if the tube is dislodged, or function 4 (doctor orders chest x‐ray) if the tube has not moved.

Once correct tube positioning is confirmed, nursing staff may commence or continue NG feeding (function 9). Pre‐conditions for this function include a prescription. Resources include nurse time and skill, access to correct feed and equipment to deliver the feed.

Once the feed is running, at some point, it will be paused (function 10). Pre‐conditions for pausing feed include prescribed feeding breaks, being nil by mouth for surgery or other interventions, or concerns about tube placement. Where planned surgical interventions are subsequently delayed or postponed to the following day, patients are at risk of prolonged periods without nutrition (time) (see Vignette C, in Table S4 for an example of this). The process returns to function 8 if/when NG feeding is planned to recommence.

This process is non‐linear. Although the first two functions occur in sequence, the process will then either proceed to the nasogastric tube placement cycle if there is no NG tube already in place (at function 3), or to the NG feed delivery process (at function 8). At any point in the delivery process, the tube may become dislodged or placement checks fail, at which point the process will return to the placement cycle—either at function 3 or 4 depending on the output of function 8 (tube displaced, or cannot confirm in stomach).

## 6 Discussion

We used a mixed methods approach to investigate the underlying reasons for, and plausible consequences of, poor nutrition delivery for post‐ICU patients, which was judged to be a contributory factor to probably avoidable deaths in our prior work. In‐depth reviews allowed us to explore the implications of failure of handover and ongoing nutrition provision, and vignettes drawn from these in‐depth reviews helped illustrate the problems identified. Staff interviews explored the context of care delivery and why problems may have occurred. Finally, the FRAM details structural processes of enteral delivery on general hospital wards.

Problems related to nutritional delivery were extensive, including failure to monitor intake, failure to escalate to specialist input, haste in removing enteral feeding tubes before adequate oral intake was established and poor management of enteral nutrition. These problems appeared to be due in part to a failure to appreciate the overall nutritional status of patients rather than reviewing their care daily.

### 6.1 Multi‐Professional Teamwork

The FRAM, informed by primary data from the REFLECT study, demonstrated the wide variety of professionals involved in providing post‐ICU nutritional support, including nurses, doctors, dietitians and specialist nutrition teams. This was reflected by the high frequency of ‘team structure’ identified as an underlying contributory human factor, demonstrating that good nutritional support relies on multidisciplinary collaboration (Table S5). This was also revealed by the FRAM, where multi‐professional working was essential to ensure timely, adequate delivery of enteral nutrition.

### 6.2 Identifying Malnutrition and Monitoring Nutritional Intake

Identifying poor nutritional intake is a key input in the FRAM, whether delivered through ward rounds, nursing assessment or dietetic review. All identification routes relied on adequate monitoring of nutritional intake to evidence the need for nutritional support. Opportunities to refer to dietetic or other specialist support were often missed, as exemplified in vignette A and other in‐depth reviews (Table S4). Staff interviews suggested sufficient monitoring was often undeliverable within current resource constraints on general wards (Table S6). Nutritional support/monitoring is a fundamental part of patient care but consumes substantial nursing time. Nurses receiving patients from ICU have described post‐ICU patients as having higher care needs than they are able to meet within their current workload [24, 25]. Consequently, nutrition delivery may not be prioritised, because the impact of poor nutrition may not be as immediately obvious as the failure to deliver other care [26]. Lack of clarity in who is responsible for identifying and, especially, escalating poor oral intake (e.g., doctors, nurses or dietitians) may also contribute to failures in recognising and acting on poor nutritional intake [26].

### 6.3 Early Removal of Feeding Tubes

Removal of feeding tubes before oral intake is established was also identified as a problem in the in‐depth reviews and staff interviews suggested this was due to a pressure to promote recovery towards discharge (Table S3). The FRAM identified the process of (re)placing enteral feeding tubes is at risk of prolonged delays, suggesting tubes should only be removed once it is clear they are no longer needed. Previous studies have identified a cultural drive to remove feeding tubes to promote oral intake and rehabilitation [26, 27]. However, studies show that patients recovering from critical illness have greater nutritional deficits when receiving oral rather than enteral feeding [26, 28, 29]. Oral intake can be further limited by nausea, vomiting and poor appetite following critical illness [29, 30]. Concerns that enteral feeding may suppress appetite have been disproved [31]. Avoiding early removal of NG tubes and continuing combination feeding may facilitate adequate nutrition until oral intake is fully established [8, 9, 10].

### 6.4 Delivery of Enteral Nutrition

In‐depth reviews identified multiple process issues with delivering enteral nutrition. Although limitations in reporting of nutrition delivery within the case notes did not allow for consistent quantification of these delays, vignette C describes large breaks in enteral feeding due to cancelled surgical interventions and issues with nasogastric tube placement are common in these cases (Table S4). The FRAM illustrates two processes where significant delays in enteral feeding can occur: the tube placement cycle and the feed delivery triangle (Figure 2). For any patient recovering from critical illness, cumulative delays can significantly impact patients' rehabilitation. The main control influencing the tube placement cycle was local policy, based on national advice and patient safety alerts [32, 33]. Radiological confirmation of tube placement was required on initial insertion, and re‐checks before restarting nasogastric feeding after a break. Initial reconfirmation of placement involved inspecting the point of entry of the tube into the nostril to confirm it had not visibly moved, and pH testing of aspirate. Where aspirate could not be obtained or the pH of the aspirate was not in range, radiological confirmation was again required. Whilst important for patient safety, these checks could incur severe delays in administration of enteral nutrition.

Other studies have also demonstrated poor delivery of nutrition in post‐ICU patients [26, 28, 34, 35, 36]. A key factor in poor enteral nutrition delivery was repeated fasting for procedures, resulting in prolonged breaks to feeding, similar to those described in vignette C. This was also found in previous studies [26, 29]. Implementation of fasting guidelines may increase the proportion of prescribed feed delivered and reduce the duration of fasting but may be hindered by unpredictable procedure timings [7, 37]. Although within‐ rather than post‐ICU, these findings are similar to those in our in‐depth reviews.

### 6.5 Strengths and Limitations

This study had several strengths. We published the protocol and registered our mixed methods project [19]. We collected data at three sites, selected to represent different sized hospitals and different post‐ICU service provision. Our mixed methods approach to collecting the primary data which informed this FRAM allowed detailed exploration of common care problems judged to have contributed to post‐ICU in‐hospital deaths. In addition to nutrition, we have similarly examined rehabilitation provision and out‐of‐hours discharge from ICU [13, 14]. The FRAM consolidated rich mixed methods data [20, 38], mapping the process of delivering enteral nutrition and identifying barriers that contributed to the poor nutritional support identified in the in‐depth reviews. The FRAM focused on enteral feeding as a key area to exemplify the processes of care, but we acknowledge this is only one aspect of a much larger dataset.

This FRAM was informed by primary data from the REFLECT study which focused on all post‐ICU ward care, with some limitations to the nutrition‐related data. The in‐depth reviews relied on clinical documentation [17], which was particularly poor for nutritional monitoring, limiting our ability to assess intake and quantify the magnitude of delays and periods without nutrition. We sought to mitigate this by including all available documentation (such as charts for food, fluid balance and prescriptions and multi‐professional notes), but this limitation is likely to have resulted in under‐estimation of nutritional problems. The number of cases reviewed in depth was relatively small at 40. However, we were still able to identify numerous nutrition problems in rich detail. Given our reliance on documentation, our sample may have under‐estimated the magnitude of post‐ICU nutrition problems. Although multi‐professional perspectives were sought through the interviews (including nurses, clinical support workers, doctors, physiotherapists and dietitians), not all staff members involved in nutrition delivery were included or reviewed the model. Future work should consider the perspectives of other stakeholders such as speech and language therapists, radiologists, porters and operations managers. Qualitative interviews provided few direct references to nutrition provision, although there was significant general discussion around the high workload associated with post‐ICU patients. Rather than a limitation, this absence highlights the lack of importance placed on nutritional support by both patients and staff.

### 6.6 Implications for Practice and Future Research

Our mixed methods approach provided insight into the structural complexity of delivering nutrition to patients discharged from ICU to a general ward, particularly when patients require enteral feeding. By using primary data from the REFLECT study [11, 19] we can make several recommendations which may contribute to the recommended goal of delivering 80%–90% of prescribed nutritional requirements for critical care survivors after ICU discharge [39]. These are as follows: close nutrition monitoring on the ward; early referral to specialist nutrition teams including dietetics; avoidance of early NG tube removal before adequate oral intake is established (which could be considered as 70% of nutritional needs as per ESPEN guidelines for commencing NG feeding [7]); and prioritisation of each step in the NG tube insertion process, to ensure timely and adequate enteral nutrition.

Despite the recognised importance of nutrition in the post‐acute period, there remain few studies examining this [40]. Bear et al. call for studies investigating nutrition and exercise together into the post‐ICU recovery period [41]. Future research could also focus on defining quality standards for post‐ICU nutrition delivery, including maximum time from NG insertion to feed initiation, proportion of time NG feed is delivered compared with prescription; and proportion of days the recommended goal of delivering 80%–90% of nutritional needs is achieved.

Finally, although our work focused on post‐ICU patients, our findings are likely to be transferrable to other cohorts of ward patients, such as those who are similarly elderly, relatively frail, physically dependent and/or have high care needs (see supplementary Table S1).

## 7 Conclusion

In our previous retrospective case record review, we identified nutrition delivery as a significant contributory factor to probably avoidable deaths, with the absence of a clear nutritional plan on ICU discharge common. The FRAM identified multiple points where delays can be incurred in the process of delivering enteral nutrition. Addressing such problems requires collaborative multi‐professional working to ensure patients receive the nutrition required to maximise their recovery from critical illness.

## Funding

This paper presents independent research funded by the National Institute for Health Research (NIHR) under its Research for Patient Benefit (RfPB) Programme (Grant Reference Number PB‐PG‐0215‐36149). This research was also supported by the National Institute for Health Research (NIHR) Oxford Biomedical Research Centre (BRC). The views expressed are those of the author(s) and not necessarily those of the NHS, the NIHR or the Department of Health.

## Ethics Statement

Ethical approval was granted in the UK by Wales REC 4 (reference 17/WA/0139) on 4 May 2017.

## Consent

The authors have nothing to report.

## Conflicts of Interest

The authors declare no conflicts of interest.

## Supporting information


**Table S1:** Structured Judgement Review patient characteristics.
**Table S2:** Definitions of nutrition problem codes.
**Table S3:** Nutritional problems in care delivery for non‐survivors and survivors identified in in‐depth reviews.
**Table S4:** Contributory human factors to identified problems in care related to nutrition from in‐depth reviews.
**Table S5:** Illustrative vignettes from in‐depth reviews.
**Table S7:** Expanded Table 2 describing functions related to radiological confirmation of nasogastric tube placement.

## Data Availability

The data that support the findings of this study are available on request from the corresponding author. The data are not publicly available due to privacy or ethical restrictions.
