# Supplementary material for: Opportunities to Improve Nutrition for Patients in Hospital After Discharge From an Intensive Care Unit: A Human Factors Analysis
Source: Nurs Crit Care. 2026 Feb 1;31(2):e70374. doi: 10.1111/nicc.70374 (PMC12862120; doi:10.1111/nicc.70374)
Supplement: Supplementary file 1 — Table S1: Structured Judgement Review patient characteristics. Table S2: Definitions of nutrition problem codes. Table S3: Nutritional problems in care delivery for non‐survivors and survivors identified in in‐depth reviews. Table S4: Contributory human factors to identified problems in care related to nutrition from in‐depth reviews. Table S5: Illustrative vignettes from in‐depth reviews. Table S7: Expanded Table 2 describing functions related to radiological confirmation of nasogastric tube placement. [file NICC-31-0-s001.docx]

**Opportunities to improve nutrition for patients in hospital after discharge from an intensive care unit: A human factors analysis.**

**Supplementary information**

**Methods for primary data collection**

***In-depth reviews***

We conducted a total of 40 in-depth reviews. We analysed the 20 cases identified as probably avoidable deaths in the structured judgement review^1,2^, and 20 cases of survivors (see table S1 for demographics). Participants were a convenience sample, recruited at the same time and through the same approach as the interviews (as described in the protocol and below^3^), and were not matched in any way to the probably avoidable deaths. Patients could opt to participate in either sub-study (interviews or case review), both or neither.

In-depth reviews were analysed using the established review framework^4^. The codes within the framework related to nutrition support were limited, with only two codes directly related to nutrition (3.10: other [nutrition] and 3.23: other [dehydration/malnutrition]). Indirect codes related to poor nutrition management included: inadequate handover (3.16) and lack of liaison with other staff (3.17). To analyse nutritional delivery in depth, we expanded the nutrition-related codes based on the wording from the original framework and our detailed knowledge of the data (see supplementary table S2 for definitions of each code). We then reanalysed the cases using these nutrition-related codes. All cases were re-analysed (20 cases where death was judged to be probably avoidable in the structured judgement review, and a convenience sample of 20 survivors, as described above). Nutrition problems were identified in 15 of the non-survivor cases and 5 of the survivor cases, and patients commonly experienced more than one problem. Table S3 presents the results of this nutrition-based re-analysis.

For each problem in care identified we also assigned a ‘contributory human factor’, from an additional established framework (the Contributory Factor Classification Framework) developed to support clinical incident investigation and used in similar previous studies^5^. This promoted further consideration of the context of care documented in the care record for each patient in nine major groups of factors (patient, task, individual staff, team, work environment, organisational/management, and institutional), and allowed identification of the underlying reasons why problems may have occurred^5^ (see table S4 for results).

The process for each in-depth review was as follows:

- An initial narrative account of care delivery was written as part of the Structured Judgement Review method^1,2^. This account was derived from the written medical record (including medical, nursing and allied health professional documentation, drug, fluid and food charts, where available).
- Each narrative account was examined in comparison with theoretical ‘problem free care’, as described by Hogan et al.^6^ to support identification of problems in care delivery (including omissions in care).
- For each problem identified, a short description was recorded on an Excel spreadsheet and given a ‘problem in care’ definition derived from the narrative.
- Each problem in care identified was then coded within the spreadsheet, using the problem in care framework developed by Vincent et al.^5^, and subsequently used in similar studies^4,6^. This consists of 53 codes within eight categories of clinical care.
- Each problem in care was also examined within the context of documented care delivery to identify any underlying human factors, using the Contributory Human Factor Classification Framework, which was also entered into to the Excel spreadsheet.

We drew anonymised vignettes from the in-depth reviews to illustrate common problems with nutritional support for patients discharged from ICU to the ward (see table S5).

***Qualitative interviews***

Interviews followed a pragmatic approach, suited to mixed methods. Interviews were conducted by two female nurses who were experienced qualitative researchers (a researcher and a clinical research facilitator). Both had training in qualitative research methods. Researcher one had no prior relationship with any participants. Researcher two worked clinically in one of the sites. A proportion of interviews at that site were conducted by researcher one to offer an ‘outsider’ perspective.

Participants were selected purposively to ensure a wide range of experiences. Potential staff participants were approached by either of researcher one, researcher two or a clinical research delivery team member. Patients were invited to participate by letter via the local post-ICU follow-up clinic. Family members were invited via the patient, where appropriate. Interviews with family members could either be held jointly with the patient, or separately, based on their preferences. All participants were informed of the goal of the research before the interview.

Interviews were held either by telephone, or face to face in a quiet room on the local hospital site, based on participant preference. Interviews were semi-structured, following a topic guide which was piloted. Only the researcher and participant(s) were present during interviews. Reflective notes were taken by the researcher during and after each interview, prompting reflection on any biases, assumptions, or external factors which may have affected the interview. Interviews were audio-recorded and transcribed verbatim. Interviews were analysed following Braun and Clarke’s six steps of thematic analysis^7^, supported by NVIVO software (see table S6 for example quotes).

A total of 30 multi-professional staff members were interviewed, including 13 ward nurses, three physiotherapists, three specialist registrars (mid-grade doctors), four foundation year doctors, a dietician, five critical care outreach/follow-up team nurses and one clinical support worker. A total of 26 patients (n=18) and family members (n=8) participated. Interviews were conducted with ten individual patients, seven patient/family member dyads (with three wives, two husbands and two daughters) and one bereaved family member (wife of the patient). The duration of interviews varied between 12 and 65 minutes (mean 35 minutes).

**REFERENCES**

1. Vollam S, Gustafson O, Young JD, Attwood B, Keating L, Watkinson P. Problems in care and avoidability of death after discharge from intensive care: a multi-centre retrospective case record review study. Crit Care 2021;25:10.
2. Hutchinson A. Using the structured judgement review method A guide for reviewers. London: 2017.
3. Vollam S, Gustafson O, Hinton L, Morgan L, Pattison N, Thomas H, et al. Protocol for a mixed-methods exploratory investigation of care following intensive care discharge: The REFLECT study. BMJ Open 2019;9. <https://doi.org/10.1136/bmjopen-2018-027838>.
4. Woloshynowych M, Neale G, Vincent C. Case record review of adverse events: a new approach. Qual Saf Health Care 2003;12:411–5.
5. Taylor-Adams S and Vincent C. Systems analysis of clinical incidents: the London protocol. Clin Risk 2004;10:211-220.
6. Hogan H, Healey F, Neale G, Thomson R, Black N, Vincent C. Learning from preventable deaths: exploring case record reviewers’ narratives using change analysis. J R Soc Med 2014;107:365–75. <https://doi.org/10.1177/0141076814532394>.
7. Braun V, Clarke V. Using thematic analysis in psychology. Qual Res Psychol 2006;3:77–101. <https://doi.org/10.1191/1478088706qp063oa>.

**Supplementary Tables**

Supplementary table S1: Structured Judgement Review patient characteristics

| **Characteristic** | **Avoidable deaths**  ***n*= 20** | **Survivors**  ***n*= 20** | **Cases with nutrition problems**  ***n*=20** |
| --- | --- | --- | --- |
| **Age median (IQR)** | 75 (62–77) | 65 (51–70) | 70 (61-75) |
| **Female n (%)** | 6 (30) | 7 (35) | 5 (25) |
| **APACHE II median (IQR)** | 21 (17–26) | 16 (14-22) | 21 (16-22) |
| **Clinical Frailty Scale*** | 4 (4-5) | 3 (2-4) | 4 (2-5) |
| **Admission diagnosis n (%)**  **Surgical**  **Medical**  **Trauma** | 11 (55)  8 (40)  1 (5) | 12 (60)  8 (40)  0 (0) | 7 (35)  13 (65)  0 (0) |
| **Type of admission n (%)**  **Emergency**  **Elective** | 16 (80)  4 (20) | 17 (85)  3 (15) | 16 (80)  4 (20) |
| **ICU LOS median (IQR)** | 4 (2–7) | 3 (2–6) | 3 (2-7) |
| **Post-ICU LOS median (IQR)** | 11 (4-19) | 16 (7-26) | 12 (9-26) |
| **Received nutrition support:**  **Enteral nutrition n (%)**  **Parenteral nutrition n (%)** | 7 (35)  3 (15) | 4 (20)  3 (15) | 10 (50)  4 (20) |

*K. Rockwood et al. A global clinical measure of fitness and frailty in elderly people. CMAJ 2005;173:489-495.

Supplementary table S2. Definitions of nutrition problem codes

| **Problems in care categories and sub-categories** | **Pre-specified definition/example** |
| --- | --- |
| **Handover** |  |
| ICU handover/optimisation (nutritional and fluid) | Failure to document nutrition requirements or dehydration on ICU written handover document |
| **Nutritional Monitoring and Escalation** |  |
| Failure to recognise and act on poor intake | Documented poor intake (through food charts, fluid balance charts, etc.) without action to remedy (e.g. dietitian referral or review documented) |
| Dehydration/fluid management | Documented concern regarding dehydration or fluid overload (e.g. in nursing or medical reviews) |
| Failure to monitor nutrition/fluid balance | Absence of food charts, fluid balance charts or other means of documenting intake (fluid intake includes all routes including oral and intravenous) while patient is receiving nutrition or fluid support (e.g. enteral feeding or intravenous fluids) or concerns about nutrition or hydration have been documented. |
| Specialist referral not made/delayed/advice not followed | Where referral documented as required, this was not made, delayed by more than 24 hours, or advice from specialist review was not implemented (e.g. medical documentation of need for dietitian referral but this is not documented and patient is not seen by dietitian within next 24 hours). |
| Blood result surveillance/supplementation | A need to monitor blood results (e.g. due to risk of refeeding syndrome) was documented but blood tests not undertaken, or blood results were available and advice to supplement electrolytes was documented but not implemented. |
| Recognising clinical problem related to nutrition | Symptoms of a clinical issue impacting nutrition were documented but not recognised as such (e.g. vomiting, not absorbing NG* feed, absence of bowel sounds) |
| Acting on clinical problem related to nutrition | Symptoms documented as above and recognised as potentially impacting nutrition but no action was taken (e.g. not administering anti-emetics if patient persistently vomiting, or not seeking alternative routes of nutrition when malabsorption recognised) |
| Escalating clinical problem related to nutrition | A potential nutrition problem has been recognised and initial steps to address have been taken but no further action has followed where this has failed (e.g. ongoing large NG* aspirates not responding to anti-emetics but no further action taken) |
| Delay in radiological investigation/review (diagnostic) | A radiological investigation has been documented as required (in relation to nutrition, e.g. chest x-ray for NG* placement, abdominal x-ray or CT), but has not been undertaken, was delayed by more than 24 hours, or documented review of the report was delayed for more than 24 hours |
| **Nutritional Support Provision** |  |
| Early cessation of enteral or total parenteral nutrition | Enteral or parenteral nutrition was ceased before an alternative route (e.g. oral intake or feeding) has been fully established – i.e. adequate nutritional needs are being met via the new route |
| Ensuring secure NG placement | NG* tube is displaced (e.g. accidentally removed during movement or length at entry to nostril noted as changed) |
| Delay in radiological investigation/review (related to NG tube placement) | 4 hour or longer delay in radiological investigation or review related to NG* placement where feeding is contingent on the result (e.g. more than 4 hours between documented need and x-ray or more than 4 hours from x-ray to documented report review and confirmation of placement |
| Escalation of nutritional support when route failed | Failure to seek an alternative route for nutrition when current route documented as failed (e.g. failure to convert from NG* feeding to parenteral nutrition) |
| Team leadership | Failure of team to communicate nutritional problems, formulate a plan and implement this plan (usually nursing documentation of issues not considered or acted on during ward rounds, e.g. nurse documents ongoing diarrhoea and vomiting but medical plan is to remove NG* tube and start oral diet) |

*NG: nasogastric

Supplementary table S3: Nutritional problems in care delivery for non-survivors and survivors identified in in-depth reviews

| **Problems in care categories and sub-categories** | **Non-survivors** | **Survivors** | **Total** |
| --- | --- | --- | --- |
|  | *(n=20 patients)*  ***n (%)*** | *(n=20 patients)*  ***n (%)*** | *(n=40 patients)*  ***n (%)*** |
| **Patients with at least 1 problem in any category** | **15 (75)** | **5 (25)** | **20 (50)** |
| **Handover** |  |  |  |
| ***Patients with at least 1 problem in category 6*** | ***6 (30)*** | ***0 (0)*** | ***6 (15)*** |
| ***Total number of problems identified*** | ***6*** | ***0*** | ***6*** |
| Sub-category:  ICU handover/optimisation (nutritional and fluid) | 6 | 0 | 6 |
| **Nutritional Monitoring and Escalation** |  |  |  |
| ***Patients with at least 1 problem in category*** | ***12 (60)*** | ***3 (15)*** | ***15 (37.5)*** |
| ***Total number of problems in category identified*** | ***51*** | ***8*** | ***59*** |
| Sub-category:  Failure to recognise and act on poor intake | 5 | 1 | 6 |
| Dehydration/fluid management | 6 | 0 | 6 |
| Failure to monitor nutrition/fluid balance | 7 | 3 | 10 |
| Specialist referral not made/delayed/advice not followed | 10 | 0 | 10 |
| Blood result surveillance/supplementation | 7 | 1 | 8 |
| Recognising clinical problem related to nutrition | 4 | 1 | 5 |
| Acting on clinical problem related to nutrition | 4 | 2 | 6 |
| Escalating clinical problem related to nutrition | 4 | 0 | 4 |
| Delay in radiological investigation/review (diagnostic) | 4 | 0 | 4 |
| **Nutritional Support Provision** |  |  |  |
| ***Patients with at least 1 problem in category*** | ***11 (55)*** | ***2 (10)*** | ***13 (32.5)*** |
| ***Total number of problems in category identified*** | ***13*** | ***3*** | ***16*** |
| Sub-category:  Early cessation of enteral or total parenteral nutrition | 3 | 0 | 3 |
| Ensuring secure NG* tube placement | 2 | 2 | 4 |
| Delay in radiological investigation/review (related to NG* tube placement) | 1 | 1 | 2 |
| Escalation of nutritional support when route failed | 4 | 0 | 4 |
| Team leadership | 3 | 0 | 3 |
| **Total problems in care identified** | **70** | **11** | **81** |

 *NG: nasogastric

Supplementary table S4: Contributory human factors to identified problems in care related to nutrition from in-depth reviews

| Contributory sub-factor* | Frequency  n (%) |
| --- | --- |
| **B TASK AND TECHNOLOGY FACTORS** | **1** |
| *B:1 Task design and clarity of structure* | *1* |
| **C INDIVIDUAL (STAFF) FACTORS** | **6** |
| *C:1 Knowledge and skills* | *2* |
| *C:2 Competence* | *4* |
| **D: TEAM FACTORS** | **61** |
| *D:1 Verbal communication* | *1* |
| *D:2 Written communication* | *2* |
| *D:3 Supervision and seeking help* | *2* |
| *D:4 Team structure* | *56* |
| **E: WORK ENVIRONMENT FACTORS** | **10** |
| *E:1 Staffing levels and skill mix* | *5* |
| *E:2 Workload and shift patterns* | *5* |
| **F: ORGANISATION AND MANAGEMENT FACTORS** | **3** |
| *F:2 Organisational structure* | *1* |
| *F:4 Safety culture and priorities* | *2* |
| **Total** | **81** |

*human contributory sub-factors from the framework not identified in this work are not presented in this table, for brevity

Supplementary table S5: Illustrative vignettes from in-depth reviews

| Case A (probably avoidable death) | | | | | | | | | | | | | | | | |
| --- | --- | --- | --- | --- | --- | --- | --- | --- | --- | --- | --- | --- | --- | --- | --- | --- |
| Original narrative | Evening discharge with dehydration not acknowledged in discharge document. Symptoms of ileus and dehydration overnight well managed by on-call foundation year doctor, including suggestion to measure lactate in morning. No reference to night events on Specialist Registrar morning ward round. Ongoing vomiting of faecal fluid, dehydration and no documented fluid or nutritional intake for 4 days. Day 2 consultant ward round requested nasogastric tube and intravenous fluid infusion removal, followed by vomiting episode with loss of consciousness and aspiration. Specialist Registrar ordered CT scan for suspected collection on day 2. No review of CT scan on day 3 ward round. reviewed by specialist surgical team in the afternoon: suspected ileus, can't rule out ischaemia. Day 4 consultant ward round - consider soft diet. Sudden deterioration on day 5. Lactate 10 with ischaemic bowel and perforation. Palliated in ICU. | | | | | | | | | | | | | | | |
| Problem in care narrative | Dehydrated on arrival on ward – not acknowledged in ICU discharge | Ongoing dehydration | | | No fluid balance or food chart for 4 days | | | Haste to remove NG* tube and intravenous fluids | | Failure to seek specialist nutritional advice | | | Delay in radiological investigation | | | Failure to recognise poor condition of patient, including plan to consider soft diet |
| Problem category | ICU handover/  optimisations | Dehydration/  Fluid balance | | | Failure to monitor nutrition/fluid balance | | | Early cessation of EN* or TPN^ | | Specialist referral not made/ delayed/ advice not followed | | | Delay to radiological investigation/review (diagnostic) | | | Failure to recognise clinical problem related to nutrition |
| Contributory factor category | Written communication | Staffing levels and skill mix | | | Team structure | | | Team structure | | Team structure | | | Staffing levels and skill mix | | | Team structure |
| Case B (probably avoidable death) | | | | | | | | | | | | | | | | |
| Original narrative | Late evening discharge before weekend. High dependency. No medical review until day 2. Ongoing problems with not absorbing NG* feed, converted to NJ^@^ following delay of 3 days between decision and NJ^@^ feed commencing. Poor surveillance throughout of electrolytes including bloods not being taken or checked, delay in prescribing pabrinex, and then not given. Oral potassium and phosphate prescribed but not given for various reasons and not converted to intravenous. Ongoing confusion regarding fluid status with intravenous frusemide, fluid restriction and oedema. TPN^ not started despite very poor nutritional intake. Suspected small bowel obstruction not investigated (no CT and not operated due to high risk). Electrolytes supplemented on day 28 with potassium of 2.9. No further assessment of electrolytes. Cardiac arrest during procedure on day 35. Readmitted to ICU and palliated. | | | | | | | | | | | | | | | |
| Problem in care narrative | Delay to NG* to NJ^@^ tube conversion led to delay in nutrition | | Oral electrolytes prescribed but not given and not escalated | | | Failure to investigate suspected bowel obstruction | | | Delay to TPN^ when enteral route failed | | | | | | Prolonged periods without electrolyte monitoring | |
| Problem category | Failure/delay to escalate nutritional support | | Blood surveillance/  electrolyte supplementation | | | Delay to radiological investigation/review (diagnostic) | | | Failure to act on clinical problem related to nutrition | | | Failure/delay to escalate nutritional support | | | Blood surveillance/  electrolyte supplementation | |
| Contributory factor category | Team structure | | Team structure | | | Team structure | | | Supervision and seeking help | | | Policy, standards and goals | | | Team structure | |
| Case C (survivor) | | | | | | | | | | | | | | | | |
| Original narrative | Evening discharge in complex, dependant patient with critical care neuropathy and high nursing care needs. No medical review on transfer. NG* feed stopped 07:00 for theatre at 13:00. No medical review until 13:00 - not for theatre. Feed stopped day 2 at midnight for theatre and replacement fluids started. Theatre in afternoon, returned to ward 19:00 with feed not restarted until 22:30. Good fluid balance chart completion and reviewed by Foundation Year doctor - large losses from wounds. Day 3 consultant ward round with clear plan. Comprehensive physiotherapy input. Speech and language team review - unsafe swallow continues. NG* tube fell out in afternoon. Delay in re-siting and four-hour delay to chest x-ray review by Foundation Year doctor. Unclear if feed restarted before NBM [nil by mouth] at midnight for theatre. Ongoing tachycardia and tachypnoea with Foundation Year doctor review at 02:00. Developed abdominal pain during day. Outreach review facilitating ICU Specialist Registrar review and readmission to ICU with perforated duodenal ulcer. Good management to ICU readmission. Only four hours of NG* feed intake over four days documented, despite large wound losses. | | | | | | | | | | | | | | | |
| Problem in care narrative | Prolonged periods without NG* feed awaiting surgical interventions | | | Delay to restarting NG* feed after surgical interventions | | | NG* tube dislodged | | | | Delay to re-siting NG* tube when dislodged | | | Delay to reviewing x-ray to confirm NG* tube placement | | |
| Problem category | Failure to recognise clinical problem related to nutrition | | | Failure to recognise and act on poor intake | | | NG* tube placement not protected (e.g. bridled) | | | | Failure to act on clinical problem related to nutrition | | | Delay in radiological intervention/review related to NG* tube placement | | |
| Contributory factor category | Verbal communication | | | Workload and shift patterns | | | Safety culture and priorities | | | | Workload and shift patterns | | | Workload and shift patterns | | |

 *NG: nasogastric^, @^NJ: nasojejunal, ^$^EN: Enteral nutrition, ^TPN : total parenteral nutrition

 Supplementary table S6: Illustrative interview quotes

| Quote 1 |
| --- |
| “. . . I think perhaps because once you put somebody on TPN [total parenteral nutrition] it’s quite… you set them back quite significantly in terms of their recovery period so I think people would hold out hoping the patient would improve and then they wouldn’t improve and eventually when they were started on TPN it would be a bit too late. So I don’t think it was through negligence as such I think it was more false optimism.”  *Foundation Year doctor, staff interview 8, site A* |
| Quote 2 |
| “The patients who are eating and you just presume they’re eating so they’re fine. But actually, its them, that maybe their appetite isn’t as good, and maybe as nurses we’re not as good at monitoring that, especially the elderly . . . it could be quite a few days before you suddenly think actually… they’re still not really eating.”  *Ward sister/charge nurse, staff interview 5, site A* |
| Quote 3 |
| “And so patients who are physiologically well leaving ICU having recovered from organ failure still have complex care requirements be they nutritional, fluid related, medication related, and not least psychological. And so you have a patient who’s recovered from their [physiological] insult and but still has complex issues.”  *ICU Specialist Registrar, staff interview 10, site A* |
| Quote 4 |
| “. . . and then you do get some that come down that are very complex that have got all sorts of needs and that’s where it’s important that you look at your staffing for the shift and you make sure that you’ve got staff that can deal with, for example they might have a PEG [permanent feeding tube] in, they might have a trache[ostomy], they might be on NIV [non-invasive ventilation] and they might be on lots of different things.”  *Ward nurse, staff interview 7, site B* |

Supplementary table S7: Expanded table 2 describing functions related to radiological confirmation of nasogastric tube placement

| ***Function 4: Doctor orders chest x-ray*** | |
| --- | --- |
| Input: | New nasogastric tube requires radiological confirmation of placement |
| Pre-conditions: | Nasogastric tube in place |
| Resources: | Access to electronic patient record  Team/on-call doctor time to order chest x-ray |
| Controls: | Local policy |
| Time: | Timeliness of feed administration to meet nutritional needs |
| Output: | Doctor has ordered chest x-ray |
| ***Function 5: Chest x-ray taken*** | |
| Input: | New nasogastric tube requires radiological confirmation of placement |
| Pre-conditions: | Doctor has ordered chest x-ray |
| Resources: | Radiology time to take x-ray  Porter time to take patient to department (optional) |
| Controls: | Local policy |
| Time: | Timeliness of feed administration to meet nutritional needs |
| Output: | Chest x-ray taken and ready for reporting on electronic patient record |
| ***Function 6: Upload chest x-ray report on EPR*** | |
| Input: | New nasogastric tube requires radiological confirmation of placement |
| Pre-conditions: | Chest x-ray has been taken |
| Resources: | Radiologist time and expertise to review and report on x-ray |
| Controls: | Local policy |
| Time: | Timeliness of feed administration to meet nutritional needs |
| Output: | Chest x-ray report available for review |
| ***Function 7: doctor reviews report*** | |
| Input: | New nasogastric tube requires radiological confirmation of placement |
| Pre-conditions: | Chest x-ray has been taken and reported |
| Resources: | Team/on-call doctor time to review x-ray report and confirm placement to nurse |
| Controls: | Local policy |
| Time: | Timeliness of feed administration to meet nutritional needs |
| Output: | Nasogastric tube placement in stomach confirmed or  Tube incorrectly placed |
